# Pills, Potions and Psychosocial Support in Pregnancy and the Post-Partum: Evaluating Interventions in a Specialist Perinatal Clinic

**DOI:** 10.1192/bjo.2024.491

**Published:** 2024-08-01

**Authors:** Sheridan McWilliam, Neeti Gupta, Nilamadhab Kar

**Affiliations:** Black Country Healthcare NHS Foundation Trust, Wolverhampton, United Kingdom

## Abstract

**Aims:**

Psychiatric illnesses are common in the perinatal period and many women are treated with psychotropic medications. Prescribing psychotropic medications often raises concern among patients and clinicians, because of a lack of information and no license to prescribe during pregnancy. This project aimed to evaluate the interventions offered in a perinatal clinic against the Perinatal College Centre for Quality Improvement standards. This included evaluating medications prescribed in the antenatal and postnatal periods; counselling regarding medication risks and benefits, provision of verbal and written information and psychosocial interventions.

**Methods:**

Data of 60 patients (30 antenatal and 30 postnatal) attending perinatal outpatient clinics covering two cities in Midlands, England, consecutively from November 1^st^ 2023 were collected from electronic clinical notes and clinic letters. Patients who did not attend their appointment were excluded.

**Results:**

The mean age of the sample was 30.3 ± 5.2 (range 19–41). Average gestational age was 6.5 ± 2.1 months (range 2.0–9.5) for antenatal women, and average postnatal duration was 6.5 ± 5.0 months (range 0.1–22.0) at the time of review. All women had psychiatric diagnosis, except one who was discharged back to primary care. The most common diagnoses were mixed anxiety and depression (38.3%), emotionally unstable personality disorder (38.3%), and postnatal depression (20%). The majority (75.0%) were prescribed psychotropic drugs. Antidepressants were prescribed in 66.7% of antenatal and 76.7% postnatal patients; most commonly prescribed overall were sertraline (33.3%) and citalopram (23.3%). Antipsychotics were prescribed in 30.0% of antenatal and 46.7% of postnatal patients. Aripiprazole and quetiapine were most commonly prescribed in the antenatal (both 13.3%) and postnatal (both 20%) periods. A larger proportion (40.0%) of women had as required medications; promethazine (20.0% vs 30.0%), diazepam (6.7% vs 13.3%) and zopiclone (3.3% vs 13.3%) were most frequently prescribed, with figures indicating prescription rates in the ante- versus postnatal period. None of the medications were prescribed above licensed limits nor met criteria for high dose antipsychotic monitoring. Verbal and written information about medications was provided in 78.3% and 35.0% of all cases respectively. Most (65.0%) women were offered psychological therapies, and of these, 69.2% received it.

**Conclusion:**

Most women in the perinatal period were prescribed psychotropic drugs, with higher proportions in the postnatal period. The findings suggested areas of improvement, such as offering written information, documenting the discussion of medication counselling, and to increase the psychotherapeutic support. It also suggests developing manualised educational interventions to improve information sharing with patients, and perinatal care.

